# Retrospect of the Medical Sciences

**Published:** 1843-02-04

**Authors:** 


LIST OF OFFICERS OF THE PHILADELPHIA MEDICAL SOCIETY.

*President.*
Prof. ROBERT M. HUSTON, M. D.

*Vice Presidents.*
Prof. GEORGE McCLELLAN, M. D.,
BENJAMIN H. COATES, M. D.

*Treasurer.*
JNO. WILTBANK, M. D.

*Corresponding Secretaries.*
ISAAC PARRISH, M. D.,
JOSEPH WARRINGTON, M. D.

*Librarian.*
N. D. BENEDICT, M. D.

*Orator.*
Prof. ROBERT M. BIRD, M. D.

*Curators.*
JAMES BRYAN, M. D.,
WM. TEMPLE CRAIGE, M. D.

*Senior Recording Secretary.*
JNO. J. REESE, M. D.

January 25th, 1843.

---

At a meeting of the Medical Society of Virginia, held on the 21st inst., the following officers were elected for the year 1843.

*President.*—ROBERT W. HAXALL, M. D.
*Senior Vice President.*—S. A. PATTERSON, M. D.
*Junior Vice President.*—SOCRATES MAUPIN, M. D.
*Recording Secretary.*—RICHARD C. AMBLER, M. D.
*Corresponding Secretary.*—FRED. MARX, M. D.
*Treasurer.*—JAMES BOLTON, M. D.
*Librarian.*—GEORGE G. MINOR, M. D.

R. C. AMBLER,
*Corresponding Secretary.*

*Richmond*, Dec. 23, 1842.

---

MEDICAL INSTITUTION OF YALE COLLEGE.

The annual course of lectures in this institution closed on Tuesday, the 17th inst., and the session of the *Committee of Examination* continued during the three following days. Present, on the part of the Connecticut Medical Society, ELIJAH MIDDLEBROOK, M. D., of Trumbull, *President;* LUTHER TICKNOR, M. D., of Salisbury, CHARLES WOODWARD, M.D., of Middletown, and ARCHIBALD WELCH, M.D., of Wetherfield; and, on the part of Yale College, Professors SILLIMAN, IVES, KNIGHT, BEERS, HOOKER, and BRONSON.

Seventeen candidates, after reading their dissertations and passing a satisfactory examination, were admitted to the *Degree of Doctor in Medicine*, by President DAY, of Yale College.

---

EQUIVALENTS OF FRENCH AND ENGLISH MEASURES.

| | | English inch. |
|---|---|---|
| The millimetre (thousandth part of a metre) | = | 0.039 or $\frac{1}{26}$ |
| The centimetre (hundredth do.) | = | 0.394 or $\frac{2}{5}$ |
| The decimetre (tenth do.) | = | 3.937 or 4 |
| The metre | = | 39.371 or nearly $39\frac{1}{3}$ inches,—say $1\frac{1}{9}$ English yard. |

The decametre, hectametre, and kilometre, are respectively 10, 100, and 1000 metres.

The French inch (ordinary measure) is a trifle longer than our own, being equivalent to 1.066 English inch.

| | | |
|---|---|---|
| The French foot | = | 12.078 |
| The French toise = 6 feet English | | 4.735 |
| | or 2 yards | 2.131 |

The anne is equal to $1\frac{1}{3}$ English yard.

As respects weights and measures of capacity:—

| | | Ounce Troy. | Grain. |
|---|---|---|---|
| The milligramme | = | 0.000032 or | $\frac{1}{66}$ |
| The centigramme | = | 0.00032 or | $\frac{1}{6}$ |
| The decigramme | = | 0.0032 or | $1\frac{1}{2}$ |
| The gramme | = | 0.032 or | $15\frac{1}{2}$ |

But we may, in general, calculate, roundly, the gramme at 15 grains, or a quarter of a drachm. There are decagrammes and hectogrammes. The kilogramme, a weight in considerable use, is equivalent to 2.68, or 2 2-3 lbs. troy, or to 2 1-5 lbs. avoirdupois.

| | | Imp. Gall. | Imp. Quart. |
|---|---|---|---|
| The litre | = | 0.22 | or about $\frac{7}{8}$ |
| The hectolitre | = | 22. | or $2\frac{3}{4}$ imp. bush. |

*London Lancet.*

---

*New Treatment for Strumous Conjunctivitis.*

BY EDWARD HOCKEN, M. D.

A clean piece of stick of the nitrate of silver, having from one to two inches exposed, should be selected. The patient's eyelids are to be closed, and put slightly on the stretch, by applying the thumb of the left hand to the eyebrow, and gently raising the skin. The nitrate of silver is then to be passed (previously moistened) two or three times over the whole surface of the upper, and subsequently the lower eyelids, smoothly and without much pressure; bringing, not the point, but the sides of the stick of lunar caustic, in contact with the skin. The object of this application is only to blacken, and not to occasion any severer effects; and it will be found that, after a short time, as soon as the nitrate has had time fully to act on the fifth nerve, it will completely relieve the intolerance of light, the lachrymation, and, what is of the most importance, the spasmodic strivings of the orbicular muscle; and hence relieve the patient from that constant irritation and pressure which maintains and aggravates the affection.

---

### PENNSYLVANIA HOSPITAL.

CLINICAL Instruction to a private class will be given at the Pennsylvania Hospital during the summer months, commencing in April, by the gentlemen having charge of the house during this period.

In the course of instruction proposed, students will be trained in the practical study of disease at the bed side during the daily visits to the wards, and also have a series of lectures delivered on some of the most important subjects presenting themselves in the practice of the house.

From April to July—Medicine—Dr. PEPPER.
" " Surgery—Dr. NORRIS.
July to Nov.—Medicine—Dr. STEWARDSON.
" " Surgery—Dr. E. PEACE.

Fee for the Course, including the privilege of attending the practice of the house for one year, . . . . . . . . . $30

For those already holding the ticket of the house, . . . . . . . . $20

Feb. 4—3t.

